# Dynamic correlation between CTL response and viral load in primary human immunodeficiency virus-1 infected Koreans

**DOI:** 10.1186/1743-422X-7-239

**Published:** 2010-09-16

**Authors:** Gab Jung Kim, Hak Sung Lee, Kee-Jong Hong, Sung Soon Kim

**Affiliations:** 1 Division of AIDS, Center for Immunology and Pathology, Korea National Institute of Health, Seoul, Korea

## Abstract

**Background:**

HIV-1 specific cytotoxic T lymphocytes (CTLs) have an important role as antiviral effector cells for controlling HIV-1 infection.

**Methods:**

To investigate CTL response during the early stage of HIV infection, we measured immunity-related factors including CD4^+ ^T cell counts, CD8^+ ^T cell counts, HIV-1 RNA viral loads and IFN-γ secretion according to CTL response in 78 selected primary HIV-1-infected Koreans.

**Results:**

The CTL response was strongly induced by HIV-1 specific Gag and Nef peptides (p = 0.016) compared with induction by Tat or Env peptides. These results suggest that the major antiviral factors inducing strong HIV-specific CTL responses are associated with the Gag and Nef viral regions in primary HIV-1 infected Koreans. The relationship between viral load and CTL response showed varying correlations with time following HIV infection. CTL response was inversely correlated with viral loads at preseroconversion stage I (r = -0.224 to -0.33) and changed to a positive correlation at the preseroconversion stage II (r = 0.132 to 0.854). Finally, it changed to an inverse correlation again after seroconversion until a viral set point was established on serological profiling (r = -0.195 to -0.407).

**Conclusions:**

These findings demonstrate a dynamic correlation between viral load and subsequent CTL responses during early HIV infection.

## Background

Human immunodeficiency virus type 1 (HIV-1) specific CD8^+ ^T cells play a key role in the control of viral replication during HIV-1 infection. The cytotoxic T lymphocyte (CTL) response is mainly measured at the early stage of infection and its appearance coincides with a rapid fall in plasma viremia during the early stage of infection with HIV-1 [1]. One of the well-characterized effector functions of CD8^+ ^T cells in the control of viremia is interferon gamma (IFN-γ) secretion. IFN-γ secreted by CD8^+ ^T cells inhibits the viral replication through induction of antiviral proteins and host immune responses that kill infected cells. Therefore, strong CTL responses are often associated with better virus control and slower disease progression during the early stage of HIV infection [2].

Analyses of epitopes or epitope-rich immunodominant regions inducing HIV-specific CTLs are likely the best way for protection against infection, and offer preferable approaches for vaccine development [3]. In particular, the HIV peptides Gag and Nef have been suggested as being more frequently recognized than Env and Pol in subjects during the early stage of HIV infection [4]. However, there have been diverse reports about the induction of specific CTL responses by HIV peptides and it is still debatable if a role of each peptide can be constantly associated with immunogenic reactions with time during the early stages of HIV infection. Some results have shown that the frequencies of IFN-γ-secreting cells during HIV infection were positively correlated with viral load [5-7], while other studies indicated that the level of IFN-γ induced by CTL responses was negatively correlated with viral load [8,9]. Furthermore, some other reports suggested that there was no significant correlation between virus-specific T cell responses and HIV-1 viral load [10-12]. Thus, the relationship between immune components and virological events in HIV-1 infection remains controversial. The factors leading to different conclusions from each research group might include varying parameters of each study population, such as ethnic differences, clinical status or the different genetic traits of HIV strains [10,13].

To understand the characteristics of HIV-specific CTL responses in the control of virus replication during the early stage of HIV infection, we investigated the correlation between HIV-1 RNA viral load and HIV-1-specific CTL responses through measurement of IFN-γ secretion in response to overlapping peptide stimulation in peripheral blood mononuclear cells (PBMCs) from Korean subjects with primary HIV infection (PHI). These constituted a homogeneous ethnic group [14] and were infected with the distinct HIV-1 Korean clade B [15].

## Methods

### Study samples

The Korean National Institute of Health (KNIH) performed final confirmation tests for the samples. These were identified as being from subjects with an indeterminate HIV status or who were suspected as having an acute HIV infection, through a hospital or local public health and environment institute (IPHE), as part of the national HIV testing strategy. Among the samples referred to KNIH for confirmation, we selected 78 subjects identified by serological testing as being at an HIV-1 preseroconversion stage or at the seroconversion stage. These patients were followed up to confirm their HIV infection status using antibody detection. The samples were analyzed for CD4^+ ^T cell counts, CD8^+ ^T cell counts and HIV RNA viral load. We collected the subjects' epidemiological data and treatment history from hospitals or public health centers. All of these patients were antiretroviral therapy naive.

### Flow cytometry analysis of CD4^+ ^and CD8^+ ^T cell subpopulations

The numbers of CD4^+ ^T cells and CD8^+ ^T cells in PBMCs were counted after blood was taken from the subjects using tubes with EDTA anticoagulant. A CD4-FITC/CD8-PE/CD3-PC5 monoclonal antibody mix (Beckman Coulter, Fullerton, CA, USA) was added to 100 μL of each specimen and incubated for 15 min at room temperature in the dark. IgG1-FITC/IgG1-PE/IgG1-PC5 (Beckman Coulter) was used as an isotype control. Red blood cells were removed using Immunoprep™ reagent lysis solution (containing 1.5% formaldehyde, Beckman Coulter) after incubation. Finally, the stained cells were analyzed using a Cytomics FC500 flow cytometry system (Beckman Coulter).

### Quantitative analysis of HIV-1 RNA

Using a Nuclisens Easy HIV-1 system (BioMerieux, Durham, NC, USA), HIV-1 RNA in each patient's plasma was quantified. Plasma was stored at -70°C after isolation from HIV-1 infected blood. After mixing aliquots of plasma (200-2000 μL) with lysis buffer (containing guanidine thiocyanate and Triton X-100, BioMerieux) by vigorous shaking for 30 min at 37°C, nucleic acids were absorbed by inverted shaking with 50 μL silica for 10 min. We used 20 μL aliquots of calibrators including the HIV-1 Gag gene (Qa, Qb, Qc) as an internal control. Pure HIV-1 RNA was isolated using NucliSens Extracter (BioMerieux.) Isolated HIV-1 RNA was amplified using the Nucleic Acid Sequence Based Amplification method and amplicons derived from a single strand HIV-1 RNA were collected from the amplified products. Collected HIV-1 amplicons were quantified using a NucliSens electrochemiluminescence reader (BioMerieux) after hybridization.

### Measurement of CTL responses using an IFN-γ enzyme-linked immunosorbent spot (ELISPOT) assay

Cryopreserved PBMCs were thawed and cultured in RPMI 1640 medium containing 10% fetal bovine serum (FBS) and 1% penicillin/streptomycin (Gibco, Grand Island, NY, USA) for 24 h at 37°C. Cultured PBMCs were counted using trypan blue vital staining (Gibco) at the beginning of the IFN-γ ELISPOT assay. IFN-γ precoated plates (Mabtech, Stockholm, Sweden) were blocked with culture medium containing 10% FBS, and 1.5 × 10^5 ^PBMCs were added to each well. Then, HIV specific peptides (Gag p17, Gag p24, Tat, Env gp120 or Nef), dissolved in DMSO with a final concentration of 5 μg/mL, were added. The concentration of DMSO was always less than 0.5% after dilution. The peptides were overlapped each other by 10 mer amino acids (from the National Institute of Biological Standards and Control, UK). Phytohemagglutinin (5 μg/mL, Sigma-Aldrich, St. Louis, MO, USA) and CD3 antigen (100 ng/mL; Mabtech) were added as positive controls. Plates were incubated under 5% CO_2 _in air at 37°C for 24 h, and developed using alkaline phosphate-conjugated monoclonal antibody 1-B6-1 and NBT/BCIP substrate (Mabtech) for 15 min at room temperature. Spot formation was analyzed using an ELISPOT Reader (Immunospot S5 Micro Analyzer, Cellular Technology Ltd., Cleveland, OH, USA). Results are expressed as spot-forming cells (SFC)/10^6 ^PBMCs = 10^6 ^× [(SFC number/well)/(number of cells/well)].

### Statistical analysis

Statistical analyses were performed using SAS software version 9.1(SAS Institute Inc., Cary, NC, USA). Spearman's rank correlation test was used to determine any correlation between the HIV-1-specific CTL response and HIV RNA viral load.

## Results

### Baseline characteristics in Korean subjects with PHI

We selected 78 subjects with PHI: 72 men and 6 women with a mean age of 35.3 years. The main transmission route of 44 subjects was recorded as sexual contact: 61% by heterosexual transmission and 39% by homosexual transmission. Of the subjects, 45 had an HIV preseroconversion status in that only antigen was detected, without any antibody at the initial tests. The other 33 individuals were identified as indeterminate by western blot testing. However, all subjects turned out to be seropositive in the follow-up tests. The mean duration between receiving the first referred sample and the follow-up sample was about 51 days (Table 1).

**Table 1:** Serological characteristics of the 78 study subjects with primary HIV-1 infection.

		Sample	
		
Characteristics		Initial sampling	Follow-up
**Serological status**			
EIA (mean, range)	Antigen ratio (OD/CO)	13.140 (0.243~30.612)	3.379 (0.063~24.793)
	Antibody ratio(OD/CO)	1.910 (0.288~14.135)	9.504 (0.159~19.608)
PA (reactivity, No.)	Reactive	20	66
	Non-reactive	58	12
WB (band pattern, No.)	Negative	45	6
	Indeterminate	29	22
	Positive	4	50

EIA, Enzyme Immunoabsorbent Assay. PA, Particle Agglutination. WB, Western Blot. OD/CO, ratio of optical density to cut-off value.

Only 50 of the 78 subjects showed positive responses in the ELISPOT assay. These CTL responders were divided into four groups according to the serological profile and the interval between the initial and follow-up visits. This was based on the detection of HIV-1 specific antigen and antibodies in plasma. In group I (preseroconversion group I, n = 7), HIV RNA viral load and HIV-1-specific enzyme immunoassay (EIA) antigen levels were extremely high and HIV-1 antibodies were weakly detectable. In group II (preseroconversion group II, n = 12), the HIV-1 specific EIA antigen value was decreased and antibodies were detectable by the EIA system and the western blot pattern was indeterminate. In group III (seroconversion group I, n = 13), HIV-1 specific EIA antigen was undetectable, antibody values were high and western blots were completely positive. Group IV consisted of 18 subjects who had also undergone seroconversion. The HIV-1 specific antigen and antibody profile of this group showed a similar pattern to group III. The follow-up durations of groups I, II and III were within 2 weeks, 1 month and 2 months of the first visit, respectively. For the follow-up samples, the mean values were 356 cells/mm^3 ^(range 129-765) for CD4^+ ^T cell count, 1,464 cells/mm^3 ^(range 406-5,937) for CD8^+ ^T cell count and log_10 _5.08 copies/mL (range 1.40-6.82) for HIV-1 RNA viral load (Table 2).

**Table 2:** Baseline characteristics of cytotoxic T lymphocyte (CTL) responses in Korean subjects with primary HIV infection

	Characteristics				
	
	Serological profile		Immunological profile		
	
Group	At initial sampling	At follow-up*	CD4^+ ^T cell (cells/mm^3^)*	CD8^+ ^T cell (cells/mm^3^)*	Viral load (log_10 _copies/mL)*
**Total** **(n = 50)**			356 (129-610)	1,464 (366-5,937)	5.09 (2.53-7.84)
I (n = 7)	EIA Ag+	EIA Ag+, EIA Ab+/-, PA+/-, WB+/-	299 (131-507)	1,322 (581-2,190)	6.08 (5.30-7.48)
II (n = 12)	EIA Ag+	EIA Ag +/-, EIA Ab+, PA+, WB+	339 (209-614)	1,547 (520-4,955)	4.91 (3.59-6.48)
III (n = 13)	EIA Ag+, EIA+/-, PA-, WB+/-	EIA Ag-, EIA Ab+, PA+, WB+	342 (129-765)	1,784 (366-5,937)	4.82 (3.40-6.20)
IV (n = 18)	EIA Ab+, PA+/-, WB+/-	EIA Ag-, EIA Ab+, PA+, WB+	393 (165-610)	1,216 (446-2,432)	5.07 (2.53-6.82)

* Measured at the time of HIV-1-specific CTL analysis

Group I (preseroconversion group I): subjects were HIV seronegative at the initial sampling time and the follow-up duration was within 2 weeks of first referral.

Group II (Preseroconversion group II): subjects were HIV seronegative at the initial sampling time and the follow-up duration was within 1 month of first referral.

Group III (Seroconversion group I): subjects were starting to become seropositive at the initial sampling time and the follow-up duration was within 2 months of the first referral.

Group IV (Seroconversion group II): subjects were HIV seropositive at the initial sampling time or starting to become seropositive.

### CTL response induced by HIV-specific peptides

**Figure 1:**
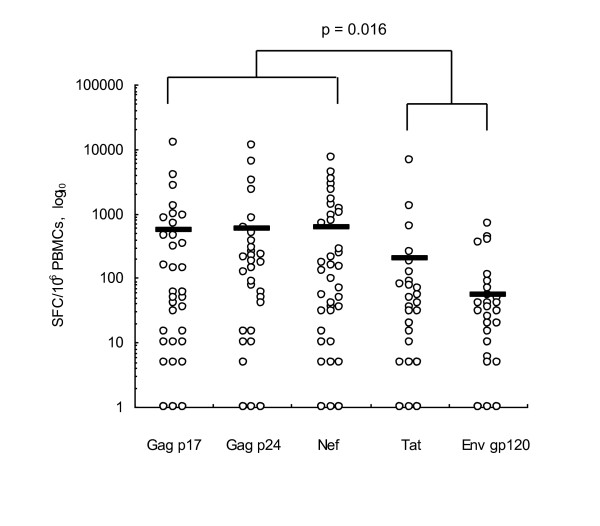
**CTL responses for HIV-1 infected Koreans**. Interferon gamma (IFN-γ) production was measured by enzyme-linked immunosorbent spot (ELISPOT) assay after treatment with overlapping peptides. Gag p17, Gag p24 and Nef induced higher levels of IFN-γ than Tat or Env gp120. The solid horizontal bars represent mean values for each group.

For the ELISPOT assay results, only 50 (64%) of these 78 subjects with newly diagnosed PHI showed IFN-γ spot formation in comparison with the positive controls. The HIV-specific CTL response in these responders was strongly induced by Gag and Nef peptides (Fig. 1). Spot formation induced by Gag p17, p24 and Nef peptides was strongly enhanced while that for Env or Tat peptide was not (p = 0.016). Cell viability reflected the effects of CTL response. The responder group showed higher cell viability than the nonresponder group in the IFN-γ ELISPOT assay: 79.6% for nonresponders and 89.2% for responders (p < 0.001; data not shown).

### Correlation between CTL response and HIV viral load

**Figure 2:**
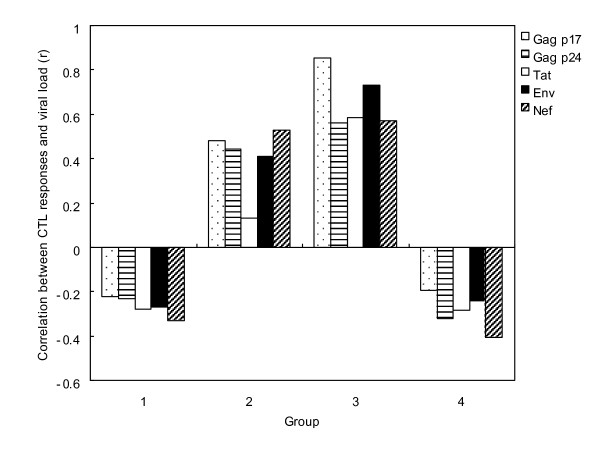
**Correlation between HIV-1 specific T cell response and plasma viral load in subjects with HIV primary infection**. Each column represents change of correlation value between HIV-1 specific CTL responses and viral load. CTL responses were performed using overlapping HIV-1 peptides: Gag p17 (spotted bars), Gag p24 (horizontal dashed bars), Tat (white bars), Env gp120 (black bars) and Nef (dashed bars).

Correlation analysis between CTL response and viral load in 50 CTL responders demonstrated that RNA viral load during the PHI period did not correlate with CTL responses (Fig. 2). HIV-specific CTL response induced by HIV specific peptides showed a slightly positive correlation with HIV RNA viral load with r = 0.153 for Gag p17 (p = 0.347), r = 0.01 for Gag p24 (p = 0.949) and r = 0.036 for Env (p = 0.827), respectively. However, there was no significant correlation between the CTL response induction by Tat or Nef peptide and RNA viral load.

Analysis of the CTL response and viral load in the four subgroups divided according to the follow-up duration demonstrated significant results based on the time course of infection. Fig. 2 shows the correlations of viral load and CTL response to each epitope in subjects divided into four groups based on their serological profile and elapsed time following HIV infection. For groups I (preseroconversion group I) and IV (seroconversion group II), the HIV-specific CTL responses to the five investigated peptides correlated inversely with the viral loads (r = -0.224 to -0.330 for group I and r = 0.195 to -0.407 for group IV). In contrast, the CTL response correlated positively with viral load in group II (preseroconversion group II; r = 0.132-0.530) and group III (seroconversion group I; r = 0.561-0.854). As mentioned above, patterns of correlation between viral load and CTL responses appeared to be transiently associated in the course of natural HIV-1 infections.

## Discussion

Studies on the CTL response have reported that CD8^+ ^T lymphocytes have an important role in controlling viral replication following PHI [2,16,17]. Generally, the HIV-specific CTL response to control HIV replication is influenced by various factors such as malfunction of immune cells affected by apoptotic events, modification of T cell surface antigens, changes in cytokine secretion, reduced expression of MHC (HLA) classes, mutation or in characteristic changes to the HIV antigen loci [18]. The characteristics of HIV-specific immune responses and the parameters of HIV infection in Asian populations including Koreans are not fully understood, although there are many reports on Caucasian and African populations [19-21]. Therefore, we investigated the relationship between HIV-specific CTL response and viral replication in these Korean subjects with PHI. Most of them were infected by a distinct strain of HIV-1 subtype B monoclade and their genetic background was comparatively homogeneous [14].

In our study, CTL responses induced by Gag p17, Gag p24 and Nef peptides were significantly higher than when induced by Env or Tat peptides (p = 0.016; Fig. 1). Many reports have demonstrated that Gag-specific T cell-mediated immune responses might be especially important to control viral load, considering the correlation between viral protein levels and CTL response in adults and children among diverse ethnic groups [22,23]. Furthermore, highly induced Nef-specific CTL responses correlated with high viral loads in the plasma [24]. These studies suggest that the HIV Gag and Nef peptides might be major factors inducing epitope-specific CTL responses in subjects with PHI. We detected HIV-specific CTL responses in only 50 of the 78 subjects in this series. The responder group demonstrated higher cell viability than the nonresponder group (89.2% vs 79.6%; p < 0.001). One of the determining factors for detection of CTL response is the composition of overlapping peptide sets. The peptides we used for the assessment of responses consisted of only five epitopes: Gag p17, Gag p24, Tat, Env and Nef. Therefore, we need to further investigate the induction capacity of other epitopes to understand the detailed mechanism of HIV-1-specific CTL response in Koreans.

Previous studies have suggested that the virus-specific CTL response developed in patients with PHI is responsible for the initial control of viral replication [2,25,26]. However, our results demonstrated that the CTL responses and viral load in Korean subjects with PHI did not show a constant correlation and there is still controversy about this correlation. Furthermore, the reasons for the differences in these findings remain unclear. Thus, we attempted to identify the reason for this controversial correlation in Koreans with PHI.

Based on our studies, one of the possible reasons for differences in the reports could be the duration following infection in subjects with PHI. We found that the HIV-specific CTL response was transiently associated with plasma viral load through successive clinical stages after HIV infection. In fact, the CTL response to HIV-specific peptides did not show obvious correlation to viral load in the 50 responders (Table 2). However, the correlation between CTL response and viral load in divided subgroups demonstrated different results based on the clinical status of the subjects. In detail, the CTL response was inversely correlated with HIV viral load in group I, presumed to be in an acute stage of infection, showing viral load abruptly rising without the production of HIV-1-specific antibody. While this correlation was changed to positive in groups II and III (identified as preseroconversion stage and initial seroconversion stage), it was negative again in group IV subjects who had undergone seroconversion. Therefore, we speculate that the CTL response is insufficiently activated to control viral replication during the preseroconversion stage. After this stage, the correlation changed from negative to positive because the CTL response was increasing to control the elevated viral load. During the period from seroconversion to the viral set point when virus concentration is maintained, the correlations between viral replication and the host immune response changed dynamically because CTL responses and viral load were linked during disease progression. That is, each individual can reveal a different correlation between CTL responses and viral load even during PHI. This implies that the clinical stage of each subject is an important factor for the HIV-1 specific CTL response to control virus replication and for its correlation with viral load. Moreover, the host's immune response might not be maintained constantly before the viral set point is established. Musey et al. also reported an alternating correlation during the early infection period before the viral set point was established within 6 months after seroconversion [27,28]. Therefore, a longitudinal study during a period between infection and viral set point should be performed to identify a pattern of alternating correlation between CTL responses and viral load in subjects with PHI.

In conclusion, we identified the Gag and Nef peptides as important HIV-1 specific CTL epitopes in regulating HIV-1 replication in this Korean population with homogeneous ethnic characteristics during PHI. We also found alternating correlations between HIV-1 viral load and HIV-1-specific CTL responses. The genetic background of the population might be an important factor for vaccine efficacy, particularly when limited epitope-specific vaccine designs are used. Thus, our results may help to improve the selection of antigen for the design of future HIV-1 vaccines in Korea.

## Competing interests

The authors declare that they have no competing interests.

## Authors' contributions

HS lee and GJ Kim carried out experiments and drafted the manuscript. KJ contributed to the revising the manuscript. SS Kim participated in the design of the study. All authors have read and approved the manuscript.

## References

[B1] Appay V Papagno L Spina CA Hansasuta P King A Jones L Ogg GS Little S McMichael AJ Rowland-Jones SL Dynamics of T cell responses in HIV infection J Immunol 2002 168 3660 3666 11907132 10.4049/jimmunol.168.7.3660

[B2] Cao J McNevin J Holte S Fink L Corey L McElrath MJ Comprehensive analysis of human immunodeficiency virus type 1 (HIV-1)-specific gamma interferon-secreting CD8+ T cells in primary HIV-1 infection J Virol 2003 77 6867 6878 10.1128/JVI.77.12.6867-6878.2003 12768006 PMC156203

[B3] Livingston BD Newman M Crimi C McKinney D Chesnut R Sette A Optimization of epitope processing enhances immunogenicity of multiepitope DNA vaccines Vaccine 2001 19 4652 4660 10.1016/S0264-410X(01)00233-X 11535313

[B4] Dalod M Dupuis M Deschemin JC Goujard C Deveau C Meyer L Ngo N Rouzioux C Guillet JG Delfraissy JF Sinet M Venet A Weak anti-HIV CD8(+) T-cell effector activity in HIV primary infection J Clin Invest 1999 104 1431 1439 10.1172/JCI7162 10562305 PMC409838

[B5] Betts MR Ambrozak DR Douek DC Bonhoeffer S Brenchleey JM Casazza JP Koup RA Picker LJ Analysis of total human immunodeficiency virus (HIV)-specific CD4(+) and CD8(+) T-cell responses: relationship to viral load in untreated HIV infection J Virol 2001 75 11983 11991 10.1128/JVI.75.24.11983-11991.2001 11711588 PMC116093

[B6] Buseyne F Chenadec JL Corre B Porrot F Burgard M Rouzioux C Blanche S Mayaux MJ Riviere Y Inverse correlation between memory Gag-specific cytotoxic T lymphocytes and viral replication in human immunodeficiency virus-infected children J Infect Dis 2002 186 1589 1596 10.1086/345482 12447734

[B7] Trabattoni D Piconi S Biasin M Rizzardini G Migliorino M Seminari E Boasso A Piacentini L Villa ML Maserati R Clerici M Granule-dependent mechanisms of lysis are defective in CD8 T cells of HIV-infected, antiretroviral therapy-treated individuals AIDS 2004 18 859 869 10.1097/00002030-200404090-00003 15060433

[B8] Patke DS Langan SJ Carruth LM Keating SM Sabundayo BP Margolick JB Quinn TC Bollinger RC Association of Gag-specific T lymphocyte responses during the early phase of human immunodeficiency virus 1 infection and lower virus load set point J Infect Dis 2002 186 1177 1180 10.1086/343811 12355372

[B9] Thakar MR Patke D Lakhashe SK Bhonge L Kulkarni SV Tripathy SP Gupte N Brookmeyer R Quinn TC Paranjape RS Bollinger RC Consistent subtype specific anti-HIV type 1 T lymphocyte responses in Indian subjects recently infected with HIV type 1 AIDS research human retroviruses 2002 18 1389 1393 10.1089/088922202320935465 12487810

[B10] Addo MM Yu XG Rathod A Cohen D Eldridge RL Strick D Johnston MN Corcoran C Wurcel AG Fitzpatrick CA Feeney ME Rodriguez WR Basgoz N Draenert R Stone DR Brander C Goulder PJR Rogenberg ES Altfeld M Walker BD Comprehensive epitope analysis of human immunodeficiency virus type 1 (HIV-1)-specific T-cell responses directed against the entire expressed HIV-1 genome demonstrate broadly directed responses, but no correlation to viral load J Virol 2003 77 2081 2092 10.1128/JVI.77.3.2081-2092.2003 12525643 PMC140965

[B11] Dalod M Dupuis M Deschemin JC Sicard D Salmon D Delfraissy JF Venet A Sinet M Guillet JG Broad, intense anti-human immunodeficiency virus (HIV) ex vivo CD8(+) responses in HIV type 1-infected patients: comparison with anti-Epstein-Barr virus responses and changes during antiretroviral therapy J Virol 1999 73 7108 7016 10438796 10.1128/jvi.73.9.7108-7116.1999 PMC104229

[B12] Gea-Banacloche JC Migueles SA Martino L Shupert WL McNeil AC Sabbaghian MS Ehler L Prussin C Stevens R Lambert L Altman J Hallahan CW Bernaldo de Quiros JCL Connors M Maintenance of large numbers of virus-specific CD8+ T cells in HIV-infected progressors and long-term nonprogressors J Immunol 2000 165 1082 1092 10878387 10.4049/jimmunol.165.2.1082

[B13] Wang S Zhuang Y Zhai S Zhao S Kang W Li X Yu XG Walker DD Altfeld MA Sun Y Association between HIV Type 1-specific T cell responses and CD4+ T cell counts or CD4+: CD8+ T cell ratios in HIV Type 1 subtype B infection in China AIDS research human retroviruses 2006 22 780 787 10.1089/aid.2006.22.780 16910834

[B14] Kim TG Han H Lim BU Kim WI Kim SM Distribution of HLA Class I alleles and haplotypes in Korea J Kor Med Sci 1993 8 180 186 10.3346/jkms.1993.8.3.180 PMC3053747 8240747

[B15] Kim YB CHO YK Monophyletic clade of HIV-1 subtype B in Korea: evolutionary pressure or single introduction ? AIDS Research Human Retroviruses 2003 19 619 623 10.1089/088922203322230996 12921094

[B16] Jin BX Bauer DE Tuttleton SE Lewin S Gettie A Blanchard J Irwin CE Safrit JT Mittler J Weinberger L Kostrikis LG Zhang L Perelson AS Ho DD Dramatic rise in plasma viremia after CD8(+) T cell depletion in simian immunodeficiency virus-infected macaques J Exp Med 1999 189 6 991 998 10.1084/jem.189.6.991 10075982 PMC2193038

[B17] Schmitz JE Kuroda MJ Santra S Sasseville VG Simon MA Lifton MA Racz P Tenner-Racz K Dalesandro M Scallon BJ Ghrayeb J Forman MA Montefiori DC Rieber EP Letvin NL Reimann KA Control of viremia in simian immunodeficiency virus infection by CD8+ lymphocytes Science 1999 283 5403 857 860 10.1126/science.283.5403.857 9933172

[B18] Gulzar N Copeland KF CD8+ T-cells: function and response to HIV infection Current HIV research 2004 2 23 37 10.2174/1570162043485077 15053338

[B19] Rinaldo C Huang XL Fan ZF Ding M Beltz L Logar A Panicali D Mazzara G Liebmann J Cottrill M Gupta P High levels of anti-Human Immunodeficiency Virus Type 1(HIV-1) memory cytotoxic T-lymphocyte activity and low viral load are associated with lack of disease in HIV-1 -infected long-term nonprogressors J Virol 1995 69 9 5838 5842 7637030 10.1128/jvi.69.9.5838-5842.1995 PMC189455

[B20] Novitsky V Gilbert P Peter T McLane MF Gaolekwe S Rybak N Thior I Marlink R Lee TH Essex M Association between virus-specific T-cell responses *and *plasma viral load in human immunodeficiency virus type 1 subtype C infection J Virol 2003 77 882 890 10.1128/JVI.77.2.882-890.2003 12502804 PMC140844

[B21] Bouscarat F Levacher-Clergeot M Dazza MC Strauss KW Giraed DM Ruggeri C Sinet M Correlation of CD8 lymphocyte activation with cellular viremia and plasma HIV RNA levels in asymptomatic patients infected by Human Immunodeficiency Virus Type 1 AIDS Res Hum Retroviroses 1996 12 1 17 24 10.1089/aid.1996.12.17 8825614

[B22] Edwards BH Bansal A Sabbaj S Bakari J Mulligan MJ Goepfert PA Magnitude of functional CD8+ T-cell responses to the gag protein of human immunodeficiency virus type 1 correlates inversely with viral load in plasma J Virol 2002 76 2298 2305 10.1128/jvi.76.5.2298-2305.2002 11836408 PMC135950

[B23] Masemola AM Mashishi TN Khowry G Bredell H Paximadis M Mathebula T Barkhan D Puren A Vardas E Colvin M Zijenah L Katzenstein D Musonda R Allen S Kumwenda N Taha T Gray G McIntyre J Karim SA Sheppard HW Gray CM HIVNET 029 Study Team Novel and promiscuous CTL epitopes in conserved regions of Gag targeted by individuals with early subtype C HIV type 1 infection from southern Africa J Immunol 2004 173 4607 4617 15383595 10.4049/jimmunol.173.7.4607

[B24] Bouscarat F Levacher M Landman R Muffat-Joly M Girard PM Saimot AG Brun-Vezinet F Sinet M Changes in blood CD8+ lymphocyte activation status and plasma HIV RNA levels during antiretroviral therapy AIDS 1998 12 1267 1273 10.1097/00002030-199811000-00007 9708405

[B25] Koup RA Safrit JT Cao Y Andrews CA McLeod G Borkowsky W Farthing C Ho DD Temporal association of cellular immune responses with the initial control of viremia in primary human immunodeficiency virus type 1 syndrom J Virol 1994 68 4650 4655 8207839 10.1128/jvi.68.7.4650-4655.1994 PMC236393

[B26] Oxenius A Price DA Trkola A Edwards C Gostick E Zhang HT Easterbrook PJ Tun T Johnson A Waters A Holmes EC Phillips RE Loss of viral control in early HIV-1 infection is temporally associated with sequencial escape from CD8+ T cell responses and decrease in HIV-1 specific CD4+ and CD8+ T cell frequencies J Infect 2004 190 713 721 10.1086/422760 15272399

[B27] Musey L Hughes J Schacker T Shea T Corey L McElrath MJ Cytotoxic-T-cell responses, viral load, and disease progression in early human immunodeficiency virus type 1 infection N Engl J Med 1997 337 18 1267 1274 10.1056/NEJM199710303371803 9345075

[B28] Mellors JW Kingsley LA Rinaldo CR Jr Todd JA Hoo BS Kokka RP Grapta P Quantitation of HIV-1 RNA in plasma predicts outcome after seroconversion Ann Intern Med 1995 122 573 579 7887550 10.7326/0003-4819-122-8-199504150-00003

